# Combinatorial effects of an epigenetic inhibitor and ionizing radiation contribute to targeted elimination of pancreatic cancer stem cell

**DOI:** 10.18632/oncotarget.21642

**Published:** 2017-10-06

**Authors:** Hyun-Mi Kwon, Eun-Jin Kang, Keunsoo Kang, Sung-Dae Kim, Kwangmo Yang, Joo Mi Yi

**Affiliations:** ^1^ Research Center, Dongnam Institute of Radiological & Medical Sciences (DIRAMS), Busan 46033, South Korea; ^2^ Department of Microbiology, Dankook Universty, Cheonan 31116, South Korea

**Keywords:** DNA methylation inhibitor, radiation, pancreatic cancer, cancer stem cell

## Abstract

Pancreatic cancer is associated with a high mortality rate, owing to de novo and acquired drug resistance, thereby leading to highly invasive and metastatic pancreatic cancer cells. Therefore, targeting pancreatic cancer stem cells (CSCs) may be a novel therapeutic strategy for the treatment of pancreatic cancer. Here, we combined a DNA methylation inhibitor (5-aza-2’-deoxycytidine; 5-aza-dC) and ionizing radiation (IR) to improve anti-cancer effects by inhibiting growth and proliferation and promoting apoptosis of pancreatic cancer cells *in vitro* and *in vivo*. Importantly, the combinatorial effect of 5-aza-dC with IR on sphere-forming pancreatic cancer cells was preferentially targeted toward CSCs through the downregulation of regulatory factors of self-renewal and CSC surface markers. We next performed the RNA sequencing to understand the underlying cellular mechanisms of the combined treatment with IR and 5-aza-dC in pancreatic cancer cells. Global transcriptome profiling indicated that the expression of the Oct4-centered transcriptional network of genes was significantly downregulated in cells with combination treatment. Our data suggested that combination treatment with DNA methylation inhibitor and IR may be a novel therapeutic strategy for pancreatic cancer. Overall, these findings support the use of epigenetic therapy in combination with radiotherapy to improve therapeutic efficacy by targeting and eradicating pancreatic CSCs.

## INTRODUCTION

Pancreatic cancer (PC) is malignancies with extremely poor prognosis with a 5-year survival rate of less than 5%, indicating that it is one of most lethal cancers [1, 2]. Despite extensive research in the field of pancreatic cancer and significant advances that have been made in the understanding of mechanisms underlying pancreatic cancer, little progress has been made in terms of treatment or early detection to improve the survival rate among pancreatic cancer patients [1].

Epigenetic regulation of transcriptional gene silencing is regulated by mechanisms such as mainly methylation of DNA, modification of histones, and alteration of nucleosome positions along with the DNA. Epigenetic events are reversible, therefore making epigenetic regulation extremely interesting from the viewpoint of developing new therapeutic approaches for cancer treatment. In the past decades, the DNMT inhibitor (5-aza-2′-deoxycytidine; 5-aza-dC) has been reported to have anticancer activities in patients with leukemia, myelodysplastic syndrome (MDS), and some other solid tumors [3, 4]. In addition, there are a few studies demonstrated that the combination of these epigenetic modifiers with conventional chemotherapy or radiotherapy might also be effective [5, 6]. Although there is growing evidence regarding strategies involving the use of substances that regulate cellular radiosensitivity to increase tumor radiosensitivity, the effectiveness of combination therapies remains limited and highly inefficient. A potential explanation for the failure of many cancer therapies might be that they do not eliminate cancer stem cells (CSCs), which are resistant to many current cancer treatments, including chemotherapy and radiotherapy, and can eventually survive to regenerate new tumors [7, 8].

CSCs are a subpopulation of cells from the bulk of tumor and it is distinguishable on the basis of exclusive ability to drive tumorigenesis, identifying that these cells play a crucial role in the relapse of the disease [9]. CSCs were first identified in leukemia and myeloma, and their existence has been validated in several solid tumors, such as cancers of the breast, glioblastoma, colon, liver, and pancreas [10–13]. A small population of cancer-initiating CSCs is potentially important because those cells may play a role in resistance to chemotherapy and radiotherapy, and they appear to be responsible for cancer recurrence after treatment even when most of the cancer cells have been killed [14]. In this regard, selective targeting of CSCs in tumors may lead to improvement in preventive diagnosis and development of novel therapeutics in cancer.

Pancreatic cancer is highly resistant to various chemotherapeutic agents and CSC markers in pancreatic cancer have been recently proposed [13]. Therefore, it is important to elucidate the mechanism of drug resistance based on the CSC model. In the present study, we established a therapeutic strategy using the DNA methylation inhibitor (5-aza-dC) in combination with ionizing radiation (IR) to increase the radiosensitivity of pancreatic cancer cells and evaluated the cellular mechanisms beyond this combinatorial effect. Additionally, this is the first report demonstrating the combination therapy of 5-aza-dC with IR and its potential effect on enhancing the response of pancreatic cancer to radiotherapy by killing cancer cells, particularly by targeting to CSCs within tumors.

## RESULTS

### Effects of 5-aza-dC on the radiosensitivity of pancreatic cancer cell lines

**Figure 1:**
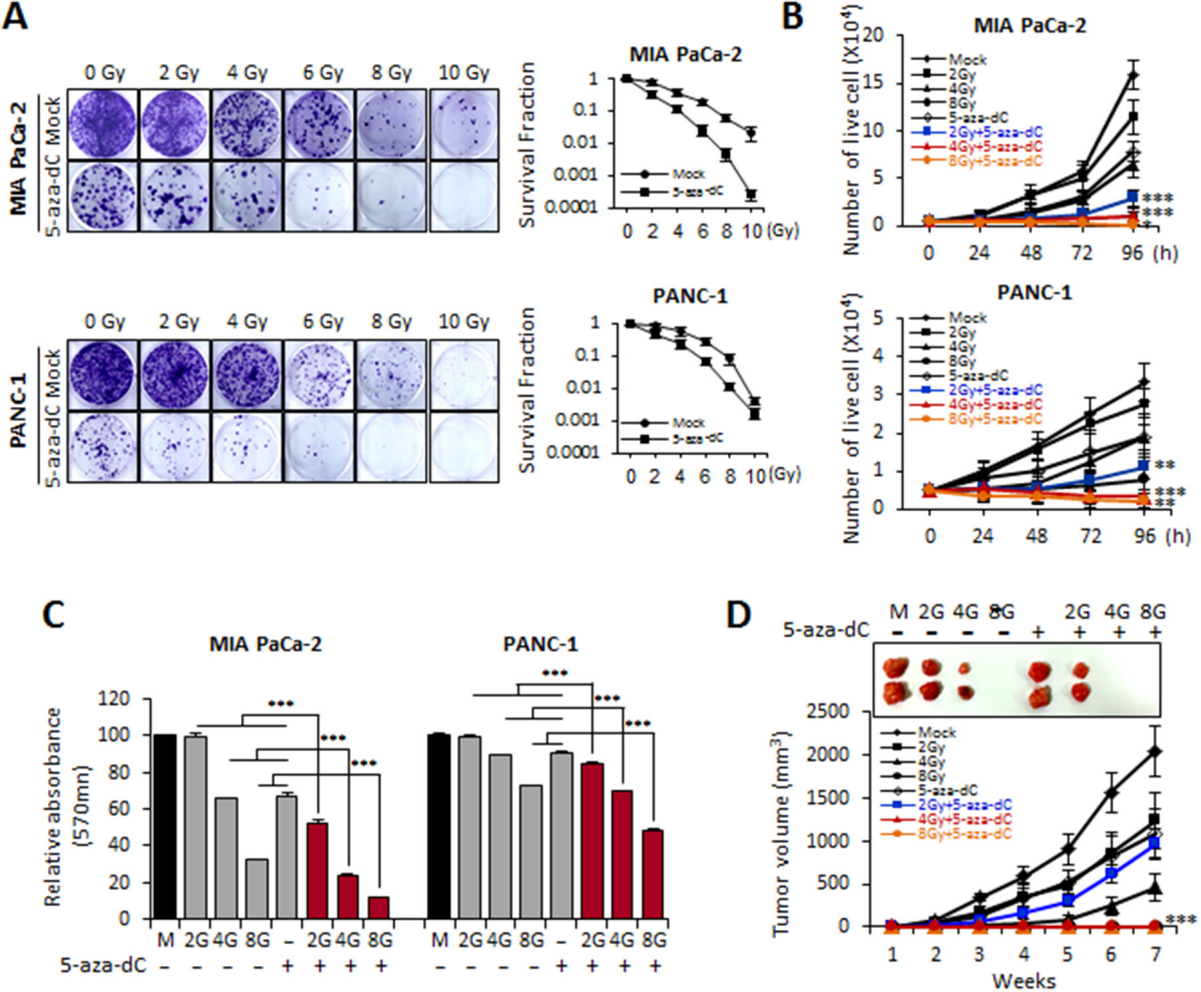
Biological effects of MIA PaCa-2 and PANC-1 cells treated with 5-aza-dC alone and with radiation **(A)** Clonogenic death of MIA PaCa-2 and PANC-1 cells treated with 5-aza-dC (5 μM) and/or IR (2, 4, 6, 8 and 10 Gy). Cells were seeded into 6-well plates (5000 cells/well) and treated with 5-aza-dC alone and combination with IR and the clonogenic survival was determined. After 2 weeks, cultures were fixed with ethanol and stained with 1.25% crystal violet. Photographs of single colonies are also shown. **(B)** Cell growth curves obtained using 5-aza-dC (5 μM) and three different radiation doses (2, 4, and 8 Gy) in MIA PaCa-2 and PANC-1 cells. **(C)** MTT assays in pancreatic cancer cells treated with 5-aza-dC (5 μM) and/or irradiation (2, 4, and 8 Gy). Data are expressed as the mean ± standard deviation of triplicate experiments. **(D)** Tumor growth following 5-aza-dC treatment alone and combination with irradiation (2, 4, and 8 Gy) in SCID mice. MIA PaCa-2 cells (1 × 10^6^ cells) that had been treated with 5-aza-dC and irradiated (2, 4, and 8 Gy) were injected subcutaneously into SCID mice (n=5, each sample group), and the average tumor size was measured once weekly for 7 weeks. Data are expressed as the mean ± standard deviation of three independent experiments. *P*-values were calculated using Student’s *t*-test. *P<0.05; **P<0.01; ***P<0.001.

First, we tested whether 5-aza-dC enhances the sensitivity of pancreatic cancer cell lines to IR. MIA PaCa-2 and PANC-1 cells were pretreated with 5-aza-dC (5 μM) for 72 hours before being exposed to different doses of IR (2, 4, 6, 8, and 10 Gy). A clonogenic assay indicated that 5-aza-dC radiosensitized the pancreatic cancer cell lines and suggested that the combination of 5-aza-dC with IR was superior to treatment with 5-aza-dC or IR alone (Figure 1A). Radiation survival curves were examined for each cell line treated with 5-aza-dC and IR after normalizing for the level of cell death induced by IR exposure alone. The survival curves for both MIA PaCa-2 and PANC-1 pancreatic cancer cell lines treated with 5-aza-dC for 24 hours before and after irradiation were steeper than the survival curves of control cells (Figure 1A). The radiosensitization caused by 5-aza-dC in pancreatic cancer cell lines was statistically significant at radiation doses higher than 4 Gy (P< 0.05).

### Combination treatment with 5-aza-dC and IR inhibits proliferation of pancreatic cancer cells *in vitro* and *in vivo*

To evaluate whether 5-aza-dC in combination with IR affects cell growth, we analyzed proliferation in MIA PaCa-2 and PANC-1 cells treated with 5-aza-dC or IR only or with a combination of both. The cell growth curves showed that cells with the combination treatment of 5-aza-dC and IR (2, 4, and 8 Gy) resulted in statistically significant growth inhibition in both cell lines at each time point examined (24, 48, 72, and 96 hours), as compared with control or in cells treated with only 5-aza-dC or IR (2, 4, and 8 Gy) (Figure 1B). In MTT assays on both cell lines, the absorbance of cells treated with a combination of 5-aza-dC and IR was significantly lower than that of cells single treatment of 5-aza-dC or IR (Figure 1C).

On the basis of our *in vitro* data showing significant growth inhibition of cells treated with the combination of 5-aza-dC and IR, we sought to examine whether tumor growth inhibition could be observed *in vivo*. MIA PaCa-2 cells were pretreated by 5-aza-dC (5 μM) for 72 hour before exposure to IR (2, 4, and 8 Gy), and then the cells were subcutaneously injected into SCID mice. Figure 1D shows the significant delay in tumor growth in response to the combination treatment with 5-aza-dC and IR (2, 4, and 8 Gy) compared with the tumor growth in the presence of single treatment with either 5-aza-dC or IR. Additionally, the size of the tumor from MIA PaCa-2 cells treated with both 5-aza-dC and IR was smaller than that of tumors established from MIA PaCa-2 cells treated with either 5-aza-dC or IR alone. Therefore, these results suggested that the combination treatment of 5-aza-dC with IR affect synergistic growth inhibition both *in vitro* and *vivo*.

### Combination treatment with 5-aza-dC and IR contributes to the induction of apoptosis in pancreatic cancer cells

**Figure 2:**
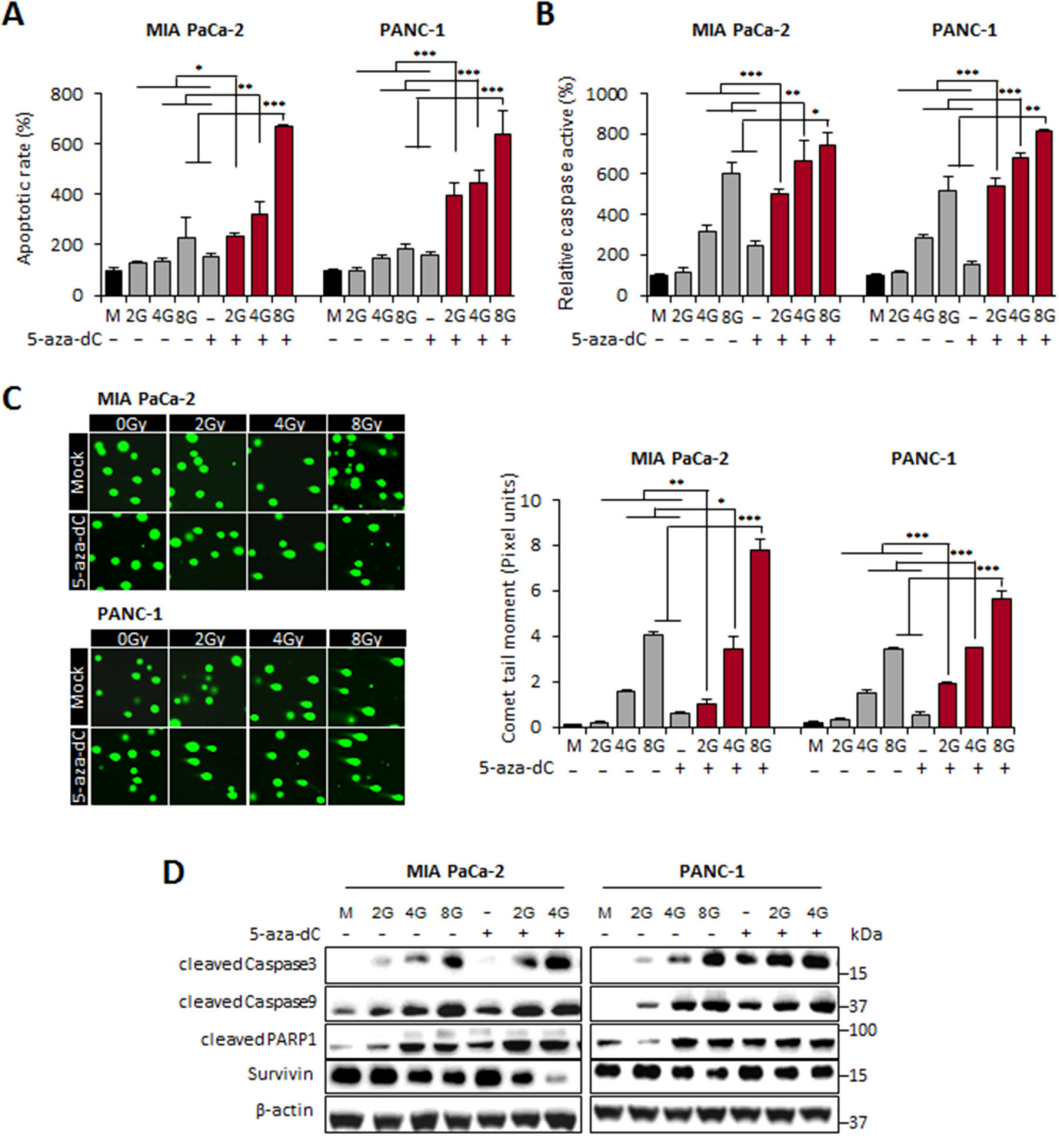
Induction of apoptosis in pancreatic cancer cells by 5-aza-dC treatment alone or with IR **(A)** The levels of apoptosis were measured using annexin V and 7-amino-actinomycin and analyzed using a FACS flow cytometer. The levels of apoptosis in MIA PaCa-2 and PANC-1 cells treated with 5-aza-dC (5 μM) alone or with irradiation (2, 4, and 8 Gy) were expressed as percentages of the total cell population at both the early and late stages of apoptosis. **(B)** The activities of caspases 3 and 7 were determined using the Caspase-Glo assay and were represented as percentages of MIA PaCa-2 and PANC-1 cells treated with 5-aza-dC alone or with irradiation (2, 4, and 8 Gy) compared with untreated cells. The graph represents data (mean ± standard deviation) from three independent experiments. **(C)** *Left:* Representative micrographs of fluorescent DNA stain using the comet assay. DNA fragmentation by the comet assay in MIA PaCa-2 and PANC-1 cells treated with 5-aza-dC alone or with irradiation (2, 4, and 8 Gy). *Right:* Quantification of DNA damaged cells represents the mean of three random microscopic fields per sample, and the error bars represent ± standard deviations. NS indicates not significant. *P*-values were calculated using Student’s *t*-test. *P<0.05; **P<0.01. **(D)** Immunoblot for the expression level of apoptosis-associated proteins (cleaved caspase 3, cleaved caspase 9, cleaved PARP1, and survivin) in pancreatic cancer cells following treatment with 5-aza-dC or IR, both alone and in combination.

To validate the enhanced induction of apoptosis by 5-aza-dC in combination with IR in pancreatic cancer cells, we investigated whether the combination of 5-aza-dC and IR (2, 4, and 8 Gy) could regulate cell apoptosis by performing flow cytometry, caspase 3 and 7 activity assays, and comet assays. The results showed that the combination of 5-aza-dC with IR significantly increased the rate of apoptosis by >3-fold or >2.5-fold in MIA PaCa-2 cells and by >1.1-fold or >1.4-fold in PANC-1 cells, as compared with that after treatment with IR or 5-aza-dC alone, respectively (Figure 2A). Because the effects of 5-aza-dC and IR on apoptosis were quantified with FACS using Annexin V/PI staining in Figure 2A, we examined the activities of caspases 3 and 7, which has been known as key effectors of apoptosis in mammalian cells, to determine whether this effect was mediated by caspases. In both cell lines, the activities of caspases 3 and 7 were markedly higher in cells treated with the combination of 5-aza-dC and IR (2, 4, and 8 Gy) than in cells treated with IR or 5-aza-dC alone (Figure 2B). Comet assay determined a similar observation with caspase 3/7 activities that cells treated with the combination of 5-aza-dC and IR showed longer tails, indicating a greater extent of DNA damage than controls and single treatment of 5-aza-dC or IR (Figure 2C). Interestingly, our immunoblot analysis revealed that the protein levels of caspases 3 and 9 were also higher in cells treated with the combination of 5-aza-dC and IR than in cells treated with either agent alone or in control cells. Cleaved PARP1, another key effector of apoptosis induction [15, 16], was also increasing in cells treated with 5-aza-dC or IR alone than in control cells. However, the protein expression level of Survivin was significantly lower in cells after combined treatment with 5-aza-dC and IR than in cells treated with each agent alone or in control cells. These findings also suggested that the downregulation of Survivin expression by the combination treatment with 5-aza-dC and IR might inhibit the survival of cancer cells and decrease cell viability. Moreover, it has been recently reported that Survivin expression positively regulates Oct4 to promote proliferation of cancer cells [17]. Therefore, we speculate that the combination of 5-aza-dC with IR might synergistically increase the level of apoptosis through the downregulation of Survivin, which is involved in regulation of Oct4, a protein that plays a critical role in CSCs.

### Alteration of CSCs or CSC-like phenotype by combination treatment with 5-aza-dC and IR through suppressing the expression of regulatory factors of self-renewal and surface markers of CSCs

**Figure 3:**
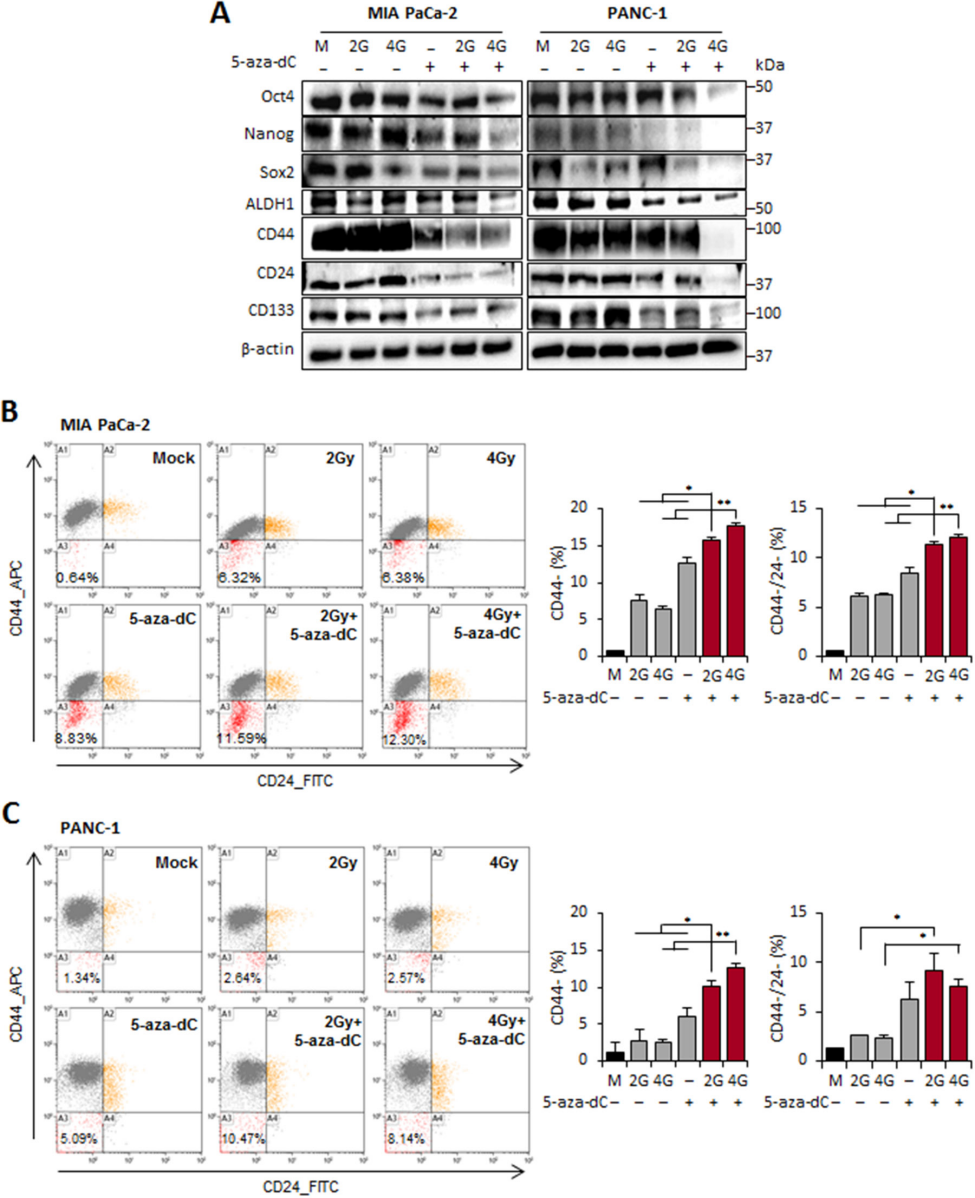
5-aza-dC treatment in combination with IR reduced the regulatory factors of self-renewal and cell surface markers of CSCs in pancreatic cancer cells **(A)** Immunoblot analysis was performed to measure the expression pattern for the regulatory factors of self-renewal (Oct4, Nanog, Sox2, and ALDH1) and cell surface markers (CD44, CD24, and CD133) in in MIA PaCa-2 and PANC-1 cells treated with 5-aza-dC alone or with irradiation (2 and 4 Gy). **(B-C)** *Right:* FACS for CD44 and CD24 cells of MIA PaCa-2 (B) and PANC-1 (C) cells treated with 5-aza-dC or IR, both alone and in combination. Orange indicated CD44+/CD24+ population. Red indicated CD44-/CD24- population. *Left:* % of CD44+, CD44- or CD44-/CD24- pancreatic cancer cells after irradiation and 5-aza-dC treatment in pancreatic cancer cells. A1, A2, A3, and A4 indicate CD44+/CD24-, CD44+/CD24+, CD44-/CD24-, CD44-/CD24+ populations, respectively. Data are means ± standard deviation from 3 independent experiments. *P*-values were calculated using Student’s *t*-test. *P<0.05; **P<0.01.

In pancreatic cancer, CSCs were first characterized as CD44+CD24+ESA+ cells with the ability to develop tumors at a higher frequency than the cells from tumor bulk.[13] CD133 has also been used to identify CSCs from both human primary pancreatic tumor tissues and cell lines [18]. We therefore examined the expression changes of regulatory factors of self-renewal such as Oct4, Sox2, and Nanog, as well as pancreatic CSC surface markers (CD44, CD24, CD133, and ALDH1) in cells treated with the combination of 5-aza-dC and IR, compared with cells treated with 5-aza-dC or IR alone or control cells, by immunoblot analysis. We observed that the levels of regulatory factors of self-renewal and surface markers of CSCs were significantly lower in cells treated with the combination of 5-aza-dC and IR. The decrease in the level of most proteins was much more pronounced in cells treated with the combination of 5-aza-dC and IR (4 Gy) than in cells treated with 5-aza-dC or IR alone (Figure 3A). To determine whether 5-aza-dC in combination with IR could inhibit the retention of stem cell-like populations, we used flow cytometry to analyze the samples on the basis of pancreatic CSC surface markers. The subpopulations of pancreatic cancer cells that express CD44 and CD24 display CSC-like properties. We found that the CD44+ or CD44+/CD24+ population decreased, and the percentage of CD44- or CD44-/CD24- population was increased in the MIA PaCa-2 cells treated with the combination of 5-aza-dC and IR, whereas the percentage of CD44+ subpopulation did not change among the 5-aza-dC, IR, and combination treatment groups (P<0.05; Figure 3B). These results were consistent with those obtained using PANC-1 cells (P<0.05; Figure 3C). Therefore, these findings demonstrated that the combination treatment with 5-aza-dC and IR target to the pancreatic CSC-like subpopulation *in vitro* by suppressing the regulatory factors of self-renewal and surface markers of CSCs.

**Figure 4:**
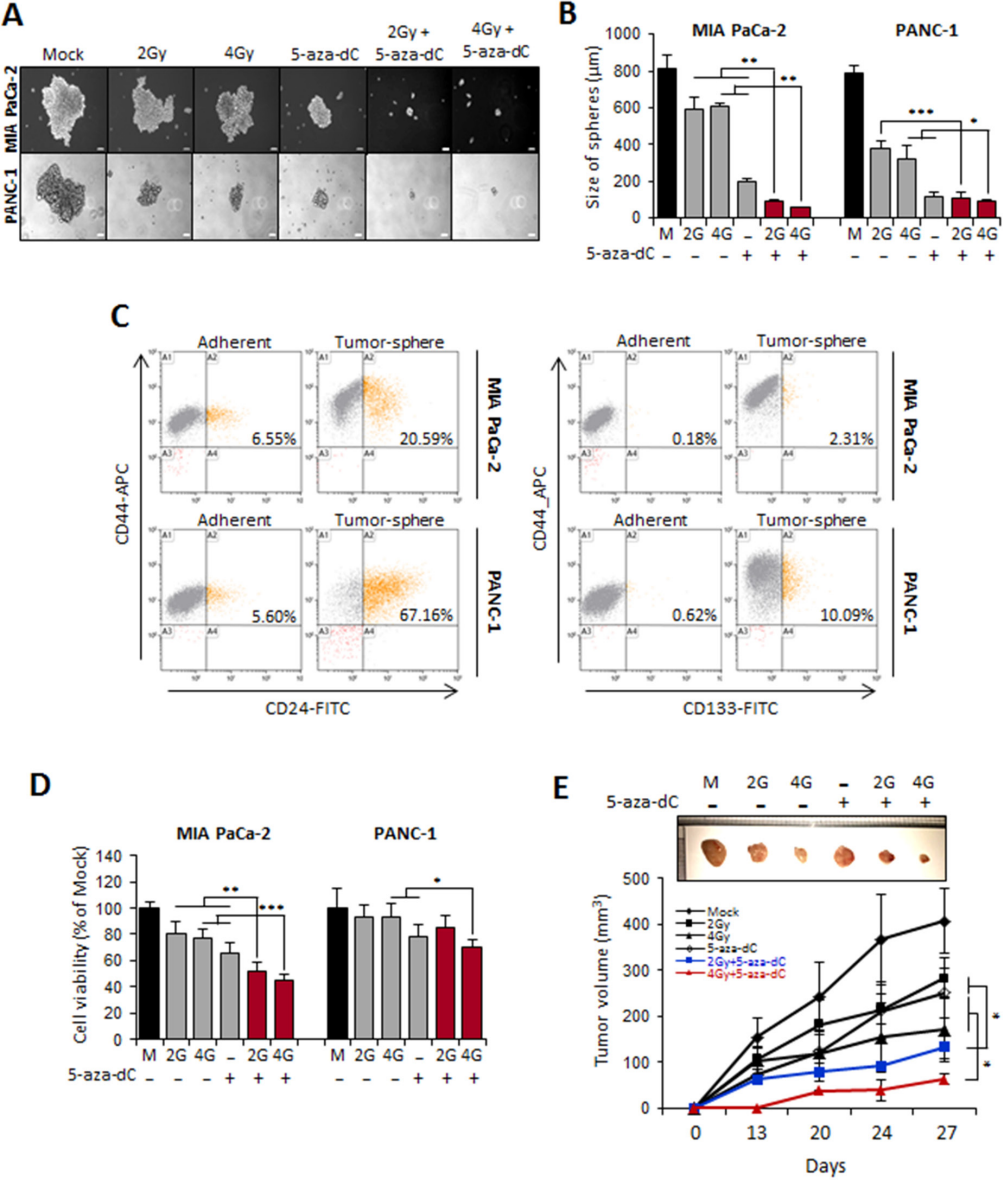
Combination treatment of 5-aza-dC with IR inhibited stem-like properties of pancreatic sphere cells **(A)** Tumor sphere formation assay of MIA PaCa-2 and PANC-1 CSCs. Cells were cultured 7 days in ultralow attachment wells with sphere media and then treated 5-aza-dC alone or with IR. Representative microscopic picture was shown. **(B)** The number of spheres with >50 um in diameter obtained from 5 x 10^3^ cells. Data are means ± standard deviation from 5 independent experiments. *P*-values between single treatment (5-aza-dC and IR) and combination of those were calculated using Student’s *t*-test. *P<0.05; **P<0.01. **(C)** % of CD44+/CD24+ between adherent and sphere forming cells from pancreatic cancer. The phenotype of CD44+/CD24- cells was measured by flow cytometry. Similar results were obtained from 3 independent experiments and representative flow cytometry pattern was displayed. **(D)** WST-1 cell proliferation assay in sphere cells treated with 5-aza-dC (5 μM) and/or irradiation (2, 4, and 8 Gy). Data are expressed as the mean ± standard deviation of triplicate experiments. **(E)** Combination treatment of 5-aza-dC and IR inhibited tumor growth *in vivo*. 1 x 10^6^ sphere cells from MIA PaCa-2 were inoculated into the SCID mice (n=5, each groups), and the average tumor size was measured once weekly for 4 weeks. 5-aza-dC significantly enhanced the anti-tumor effect of IR in sphere cells from MIA PaCa-2 xenograft model. *P*-values were calculated using Student’s *t*-test. *P<0.05; **P<0.01; ***P<0.001.

We next examined the effect of combination of 5-aza-dC in with IR on the self-renewal capacity of CSCs or CSC-like cells by conducting sphere formation assays in MIA PaCa-2 and PANC-1 cells. When 1,000 MIA PaCa-2 and PANC-1 cells were incubated for 7 days in 5-aza-dC- and IR-free sphere growth medium, most of the spheres formed had a diameter > 200 μm, whereas when the cells were incubated with 5-aza-dC or IR, spheres with diameters less than > 100 μm were formed (Figure 4A). We found that the sizes of the spheres formed after incubation with the combination of 5-aza-dC and IR were clearly smaller (less than 2 folds) than those after treatment with 5-aza-dC or IR alone or with control medium (Figure 4B). Pancreatic CSCs can be isolated by using the CD24+CD44+ESA+ phenotype [13]. Moreover, the CD133+ population is enriched in pancreatic CSCs [18]. Therefore, cell phenotypes were assessed by FACS to detect the expression of CD24+CD44+ or CD133+ (Figure 4C). As shown in Figure 4C, we observed an increase in the CD24+CD44+ subpopulation in cells isolated from spheres (20.59% for MIA PaCa-2 cells and 67.16% for PANC-1 cells) compared with adherent cells (6.55% for MIA PaCa-2 cells and 5.6% for PANC-1 cells). The CD44+CD133+ subpopulation was consistently more enriched in sphere cultures (2.31% for MIA PaCa-2 cells and 10.09% for PANC-1 cells) than in adherent cultures (0.18% for MIA PaCa-2 cells and 0.62% for PANC-1 cells). Therefore, we confirmed that pancreatic tumor spheres are enriched in CSC populations, unlike cells grown under adherent conditions. We next tested the effect of 5-aza-dC in combination with IR in our tumor sphere culture system both *in vitro* and *in vivo*. Interestingly, the combination treatment with 5-aza-dC and IR (2 Gy or 4 Gy) showed a significant decrease in tumor sphere growth (Figure 4D). In addition, we examined whether combination treatment of cells from pancreatic tumor spheres with 5-aza-dC and IR inhibited pancreatic tumor growth *in vivo*, because tumorigenic capacity *in vivo* is considered as a characteristic of CSCs [19]. Tumor sphere cells were pretreated with 5-aza-dC and IR and then subcutaneously injected into SCID mice. The data showed that the growth rate of xenografts derived from sphere-forming MIA PaCa-2 cells was slower than that of xenografts derived from cells from single agent-treated (5-aza-dC or IR with 2 Gy or 4 Gy) and control groups (Figure 4E). These results suggested that 5-aza-dC treatment in combination with IR in pancreatic sphere-forming cells was able to decrease xenograft tumor growth.

### Combination treatment with 5-aza-dC and IR can deplete pancreatic CSCs or CSC-like cells

**Figure 5:**
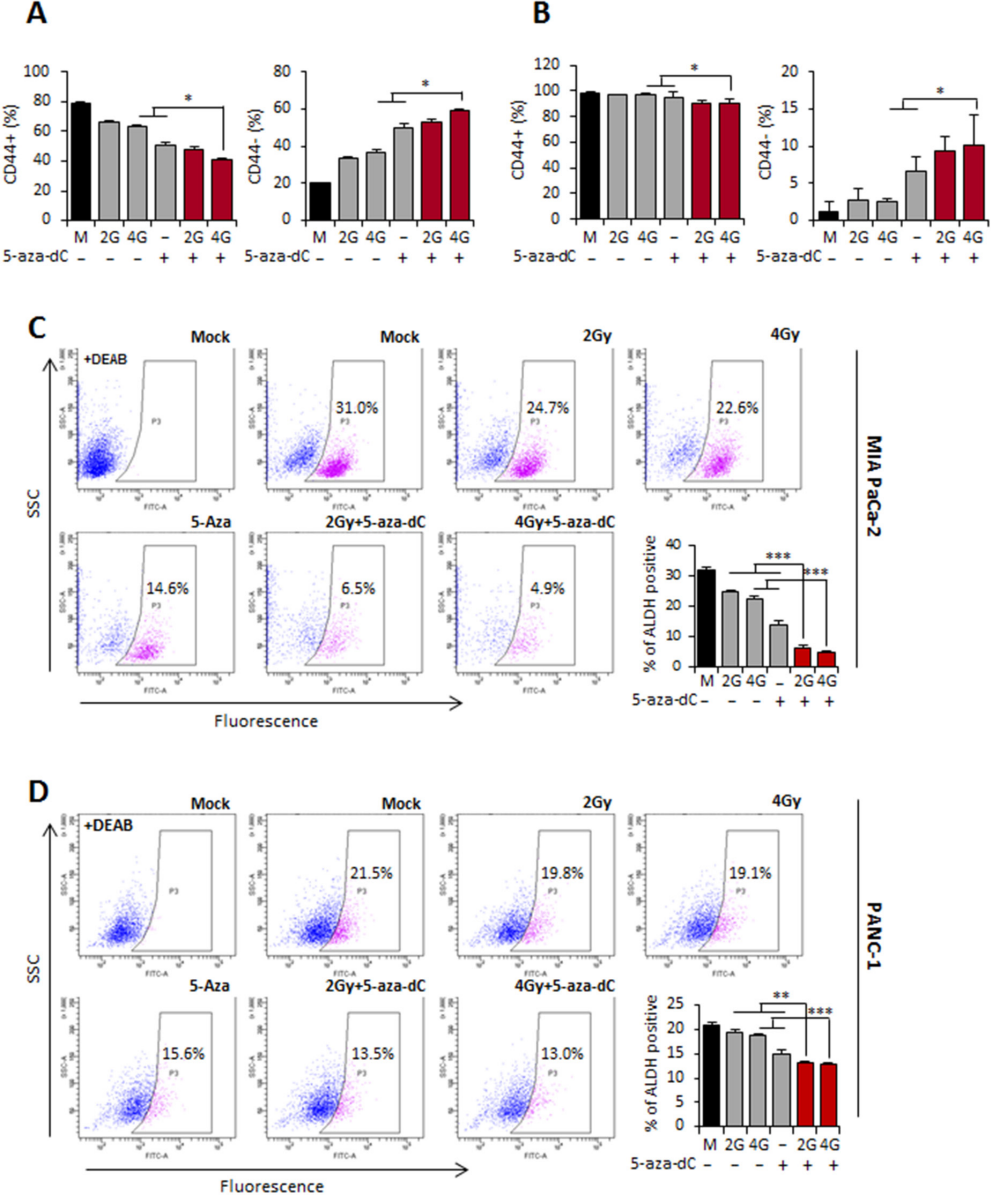
Combination treatment with 5-aza-dC and IR can deplete pancreatic CSCs **(A-B)** % f CD44+ and CD44- sphere cultured MIA PaCa-2. (A) and PANC-1 (B) in the surviving cells after treatment 5-aza-dC or with IR based on FACS. Data are means ± SD from 3 independent experiments. DEAB indicates diemethylamino-benzaldehyde was used to control for background fluorescence. *P*-values were calculated using Student’s *t*-test. *P<0.05; **P<0.01. **(C-D)** FACS for ALDH1-positive cells of sphere cultured MIA PaCa-2 (C) and PANC-1 (D) cells treated with 5-aza-dC or IR alone and combination of both. Bar graph indicated the percentage of ALDH1-positive cells of sphere cultured MIA PaCa-2 and PANC-1. Data are means ± standard deviation from 3 independent experiments. *P*-values were calculated using Student’s *t*-test. *P<0.05; **P<0.01.

As shown in Figure 4C, pancreatic CSCs were characterized as being CD44+ or CD44+/CD24+. In our analysis, although the percentage of CD44+ subpopulations of MIA PaCa-2 or PANC-1 cells isolated from tumor spheres was lower in the groups treated with the combination of 5-aza-dC and IR, the percentage of CD44- subpopulations, which lack stem cell-like properties, was significantly higher than that in the groups treated with 5-aza-dC or IR alone or the control groups (Figure 5A and 5B). These data suggested that the combination treatment with 5-aza-dC and IR was effective in attenuating the pancreatic CSC-like populations by altering the CSC or CSC-like cell phenotypes.

It has been shown that CSCs in many cancer types display high levels of ALDH1 activity, and cells with these properties are usually enriched in tumorigenic stem cells [20]. We therefore analyzed the level of ALDH1-positive subpopulations to confirm that 5-aza-dC in combination with IR decreased pancreatic CSC or CSC-like populations. Interestingly, the proportion of ALDH-positive cells in MIA PaCa-2 cells derived from tumor spheres was initially 31% and decreased to 6.5% and 4.9% in response to 5-aza-dC in combination with IR (2 Gy and 4 Gy), respectively. The average result from 3 experiments in MIA PaCa-2 cells is shown in a bar graph in Figure 5C. Similar results were obtained for PANC-1 cells derived from tumor spheres in which the ALDH-positive fraction was initially 21.5%, and it decreased to 13.5% and 13% in response to 5-aza-dC in combination with 2 Gy and 4 Gy IR, respectively (Figure 5D). These results strongly suggest that 5-aza-dC in combination with IR is more effective in attenuating pancreatic CSCs or CSC-like populations.

### Molecular alterations by the combination of 5-aza-dC with IR can affect *in vitro* sphere-forming capacity

**Figure 6:**
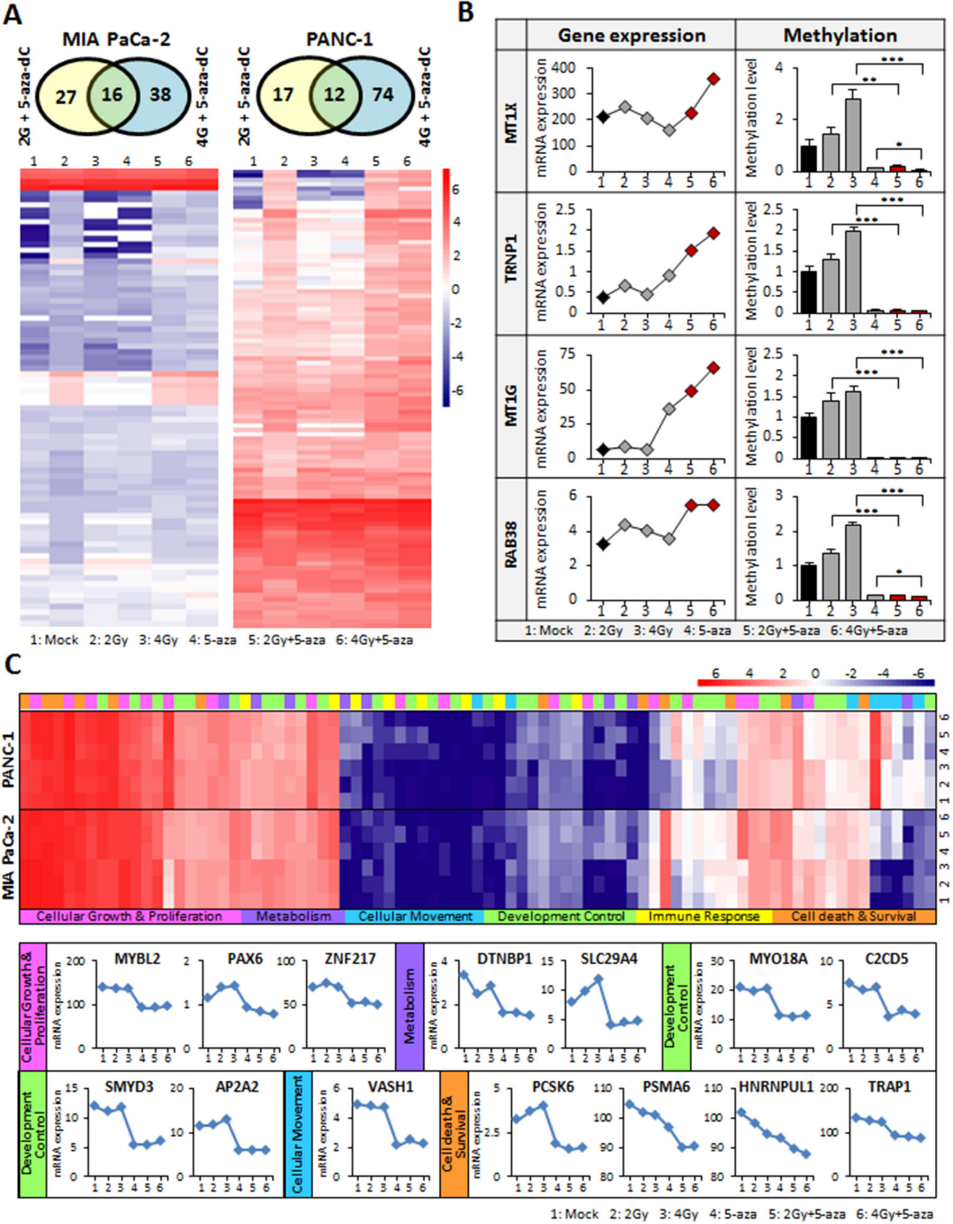
Global transcriptome analysis in pancreatic cancer cells using RNA-seq **(A)** Venn diagram for candidate genes are up-regulated by both 5-aza-dC or IR, and combination in pancreatic cancer cell lines. Heatmaps display the differential gene expression patterns (81 genes for MIA PaCa-2 cell and 103 genes for PANC-1 cell). Red represents elevated expression while blue represents decreased expression, compared with the row mean. **(B)** Negative correlation between transcriptional expression and DNA promoter methylation of best candidate genes (*MT1X, TRNP1, MT1G*, and *RAB3B*). These genes are significantly up-regulated in MIA PaCa-2 cells treated with 5-aza-dC in combination with IR (indicated red dot) compared with single treatment or control (Left panel). The methylation level is down in their CpG islands of promoter region. **(C)** Differential expression profile of Oct4 target genes (83 genes) in pancreatic cancer cells treated with 5-aza-dC alone or with IR. Summary of the functional categories of Oct4-centered transcriptional network analyzed by Ingenuity-IPA software program [27]. 14 genes showed significantly downregulated in transcriptome profile from pancreatic cancer cells treated with 5-aza-dC alone or with IR along with Oct4 downregulation. *12 genes (MYBL2, PAX6, ZNF217, DTNBP1 SLC29A4, VASH1, SMYD3, AP2A2, PCSK6, PSMA6, HNRNPUL1 and TRAP1) were from MIA PaCa-2 data and 2 genes (MYO18A and C2CD5) were from PANC-1 data*. Samples were described as numbers as following 1;Mock, 2; 2 Gy, 3; 4 Gy, 4; 5-aza-dC, 5; 5-aza-dC+2 Gy, 6; 5-aza-dC+4 Gy.

To identify potential molecular targets responsible for the anti-tumor effect of the combination of 5-aza-dC and IR, we performed comparative global gene expression analysis by RNA-sequencing (RNA-seq) in MIA PaCa-2 and PANC-1 cells treated with 5-aza-dC or IR (2 Gy and 4 Gy) or their combination and then analyzed the genes that were differentially expressed. It is well established that genes upregulated by 5-aza-dC are usually silenced by promoter hypermethylation [21]. In our analysis, we focused on candidate genes synergistically upregulated by the combination of 5-aza-dC and IR. Indeed, we identified the genes that were upregulated in response to both 2 Gy and 4 Gy IR together with 5-aza-dC and displayed their differential expression in MIA PaCa-2 (81 genes) and PANC-1 (103 genes) cells (Figure 6A and Supplementary Table 2). We next sorted out genes that showed >3-fold upregulation in response to the combination of 5-aza-dC and IR in RNA-seq profiles and then eliminated non-coding RNAs. To examine the correlation between the upregulation of gene expression and changes in promoter DNA methylation, we tested 17 candidate genes by methylation analysis. Finally, from gene expression profiles, we found that 4 genes (*MTX1, TRNP1, MT1G*, and *RAB38*) showed strong correlation between de-methylation by 5-aza-dC or 5-aza-dC in combination with IR (2 Gy and 4 Gy) and upregulation in response to the combination treatment (Figure 6B).

As shown in Figure 3A, we observed that the level of Oct4 expression was significantly decreased in cells treated with the combination of 5-aza-dC and IR. Oct4 modulates a series of signaling pathways, such as the Wnt/b-catenin, TGF-b, JAK-STAT3 signal pathways, thereby activating or inhibiting downstream target genes [22, 23]. Thus, we searched for genome-wide alterations in the expression of Oct4-bound genes involved in various cellular functions related to tumorigenesis, such as cellular growth, proliferation, and movement and cell death and survival, as well as developmental processes [24]. To test the alterations in global gene expression of Oct4 target genes, we screened for the target genes of Oct4 and analyzed the changes in their expression patterns in our transcriptome profiles (Figure 6C). Interestingly, 16 genes (*MYBL2, PAX6, ZNF217, DTNBP1, SLC29A4, MYO18A, C2CD5, SMYD3, AP2A2, VASH1, PCSK6, PSMA6, HNRNPUL1*, and *TRAP1*) exhibited significantly decreased expression in cells treated with 5-aza-dC in combination with IR compared with single agent-treated or untreated control cells, a result consistent with the level of Oct4, as shown in Figure 3A. Our data suggested that the combination treatment with 5-aza-dC and IR in pancreatic cancer cells decreases the sphere-forming capacity associated with the global downregulation of Oct4-centered network of genes.

## DISCUSSION

This study found that a DNA methylation inhibitor, i.e., an epigenetic modifier, in combination with radiation is an efficient therapeutic approach to kill pancreatic cancer cells and importantly that the combination of an epigenetic drug with radiotherapy is able to target pancreatic CSCs or CSC-like cells. Moreover, 5-aza-dC increased the radiosensitivity of pancreatic cancer cells both *in vitro* and *in vivo*, thus suggesting that 5-aza-dC may potentially be useful to enhance the efficacy of radiotherapy in pancreatic cancer.

Radiotherapy is widely used as a standard treatment for many types of cancer and is frequently used as primary or adjuvant therapy in combination with surgery or chemotherapy or both. The DNA methylation inhibitor (5-aza-dC) has been known to have little effect in solid tumors as a single agent [25], and little is known about the molecular mechanisms of the cellular radiosensitivity induced by epigenetic inhibitors. In this context, it is particularly interesting that combining epigenetic drugs with radiotherapy improves the therapeutic efficacy both *in vitro* and *in vivo* in several solid tumors [26–29]. Our results indicated that this effect is mediated by the induction of apoptosis, which has previously been regarded as a potential mechanism for radiosensitization [27, 30]. Additionally, our data suggest that 5-aza-dC in combination with IR is capable of increasing apoptosis in pancreatic cancer cells.

In the clinic, even though technical advances in radiation and chemotherapy have improved patient survival, treatment resistance from radio- and chemotherapy is still a prior challenge in both cancer research and treatment [31, 32]. Classical therapeutic approaches including radiotherapy or chemotherapy usually target only proliferating cells [33]. In recent years, it has been demonstrated that tumors are composed of different cell populations, among which a small subset display stem cell-like properties. Recent evidence has suggested the existence of CSC populations containing tumorigenic stem cells responsible for tumor initiation, progression, and metastasis. It has been also reported that the epigenetic agents, histone deacetylase inhibitors (HDACi) affect CSC differentiation. HDACi are used as differentiation therapy in several hematologic malignances and have also been proposed for suppressing various CSC-like properties of CD44+ enriched cells in breast and head and neck cancer [34, 35]. In this regard, we hypothesize that the combination treatment with a DNA methylation inhibitor and ionizing radiation may be efficacious in targeting CSC subpopulations in pancreatic tumors.

Pancreatic CSCs were first identified as a highly tumorigenic CD44+/CD24+/EpCAM+ subpopulation in xenografts in immunodeficient mice [13]. Hermann *et al.* have shown that the expression of CD133 in freshly isolated primary human pancreatic cancer cells can be used to identify cells with the capacity of self-renewal, sphere formation, and most importantly, *in vivo* tumorigenesis [18]. In our study, we characterized two pancreatic cancer cell lines (MIA PaCa-2 and PANC-1), using the expression of reported CSC surface markers including CD44, CD24, ESA, and CD133. However, it has been reported that CD44+ CSCs can proliferate and reconstitute the population of resistant cells. Moreover, clinical data have shown that patients with CD44+ pancreatic cancer have poor prognosis [36]. In this study, we observed that the size of the CD44+ population was significantly smaller in cells treated with 5-aza-dC in combination with IR, thus showing that the proportion of the CD44-negative population was significantly higher in cells treated with 5-aza-dC in combination with IR than in cells treated with either therapy alone (Figures 3 and 5). These data enabled us to conclude that the combination of 5-aza-dC and IR not only kills highly proliferative cells but also targets CSCs or CSC-like cells by altering their CSC-like phenotypes.

Given that ALDH1-positive cells are putative CSCs or CSC-like cells and that side population (SP) cells are also putative CSCs in many different cancer cell lines [37–39], the decrease in the proportion of ALDH1-positive cells (Figure 5C and 5D) and SP cells (Supplementary Figure 1) strongly supports that the combination treatment with 5-aza-dC and IR is effective in eliminating pancreatic CSCs or CSC-like cells.

Recent studies have demonstrated that the one of metastatic way termed epithelial to mesenchymal transition (EMT) is associated with characteristics of CSCs [40, 41]. We observed that the combination treatment with 5-aza-dC and IR significantly inhibit pancreatic cancer cell migration and invasion in both adherent and sphere-forming cells (Supplementary Figure 2A). In addition, our data suggested that treatment with 5-aza-dC in combination with IR contributes to eliminating CSCs *in vitro* and *in vivo*. We also observed that cells treated with 5-aza-dC in combination with IR had significantly decreased levels of EMT-associated markers such as N-Cadherin whereas increasing E-cadherin expression level, thus strongly suggesting that the combination treatment with 5-aza-dC and IR preferentially targets CSC populations (Supplementary Figure 2B). Together, these findings suggest that the combination treatment with 5-aza-dC and IR depletes ALDH-positive and SP cells enriched in CSCs. Additionally, we validated an intriguing regulatory mechanism in which the combination treatment alters CSC-associated phenotypes such as EMT.

Survivin is an inhibitor of apoptosis protein family, and it plays an important role in cancer progression by inhibiting cell apoptosis, regulating cell division, and inducing angiogenesis [42]. Knockdown of Survivin expression in a number of human cancer cell lines results in a significant decrease in cell growth, and combination of Survivin silencing with chemotherapeutic agents is a main approach in cancer treatment along with enhanced anti-tumor efficacy [43, 44]. In our study, treatment of MIA PaCa-2 and PANC-1 cells with 5-aza-dC in combination with IR (2 Gy and 4 Gy) significantly decreased Survivin expression (Figure 2D). Moreover, we observed that the protein level of Survivin was decreased in response to treatment with 5-aza-dC in combination with 8 Gy radiation in a dose-dependent manner (data not shown). Recently, it has been reported that Survivin expression is positively associated with the expression of the self-renewal regulatory factor Oct4 [17], in agreement with our results, thus indicating that the downregulation of the survivin-Oct4 axis may affect CSC maintenance. Interestingly, we observed that the expression levels of several regulatory factors of self-renewal and surface markers of CSCs were significantly decreased in pancreatic cancer cells treated with 5-aza-dC in combination with IR, as compared with cells subjected to either treatment alone (Figure 3A).

The transcriptome covers all RNA molecules including non-coding RNAs, mRNAs, and other small RNAs in a cell. Transcriptome analysis by next-generation sequencing (RNA-seq) allows variety studies of a transcriptome at incomparable methods. One of benefits is that RNA-seq allows for sequencing readout quantification of the transcriptome by a high throughput approach and is independent on previous investigation [45]. 5-aza-dC is a strong inducer of DNA de-methylation. The depletion of methyltransferases in the cell results in global de-methylation, which reactivates epigenetically silenced genes [21]. We have previously reported that radiation induces global hypomethylation in colon cancer cells and have identified several candidate genes whose expression is epigenetically regulated by promoter methylation [46]. In this study, we also identified four candidate genes (*MTX1, TRNP1, MT1G* and *RAB38*) that were upregulated by both 5-aza-dC and IR, and this upregulation was correlated with DNA hypomethylation at their promoter regions (Figure 6B). Although we advocate for the clinical application of this combinatorial approach, further study is necessary to understand these genes as therapeutic targets or prognosis biomarkers in pancreatic cancer.

Octamer 4 (Oct4), a POU family transcription factor, is specifically expressed in embryonic stem cells and tumor cells but not in cells from differentiated tissues [47]. In recent years, Oct4 transcript has been consistently detected in testicular germ cell tumors, seminomas, and bladder carcinomas [48, 49]. Oct4 expression has been also studied in human breast cancer stem-like cells, thus suggesting that its expression may be associated with self-renewal of CSCs and tumor formation through the activation of its downstream genes [50]. A previous study in a lung cancer model system has provided mechanistic insights into the Oct4-driven transcriptional network in the promotion of drug resistance and metastasis [24]. In terms of the loss of Oct4 expression in response to the combination of 5-aza-dC and IR in pancreatic cancer cells, we wondered whether the expression of the downstream target genes of Oct4 might also be changed. We therefore analyzed the expression pattern of these genes in the Oct4-driven transcriptional network (83 genes) from the transcriptome profiles in our study. Interestingly, dynamic gene expression changes were displayed in Figure 6C along with biological signaling pathway categorization. Finally, fourteen genes (*MYBL2, PAX6, ZNF217, DTNBP1, SLC29A4, MYO18A, C2CD5, SMYD3, AP2A2, VASH1, PCSK6, PSMA6, HNRNPUL1*, and *TRAP1*) exhibited significant decreases in response to the combination treatment with 5-aza-dC and IR in our transcriptome profiles by RNA-seq (Figure 6C). Thus, we provided the first demonstration of genome-wide changes in the OCT4-regulated transcriptional network in response to treatment with an epigenetic inhibitor in combination with IR in pancreatic cancer cells, on the basis of RNA-seq technology. Tang and colleagues have demonstrated that Oct4 inhibits tumor suppressor genes such as *PTEN*, whereas Oct4 activates oncogenes, such as *Tenascin-C* (*TNC*), through differential interactions with the transcription factor Sp1. In addition, the Oct4-mediated downregulation of tumor suppressor genes contributes to drug resistance. In this regard, our data strongly supported that downregulation or lack of Oct4 expression by treatment with 5-aza-dC in combination with IR might activate tumor suppressors and inhibit oncogenes, thus resulting in the interference with tumor development *in vitro* and *in vivo*.

**Figure 7:**
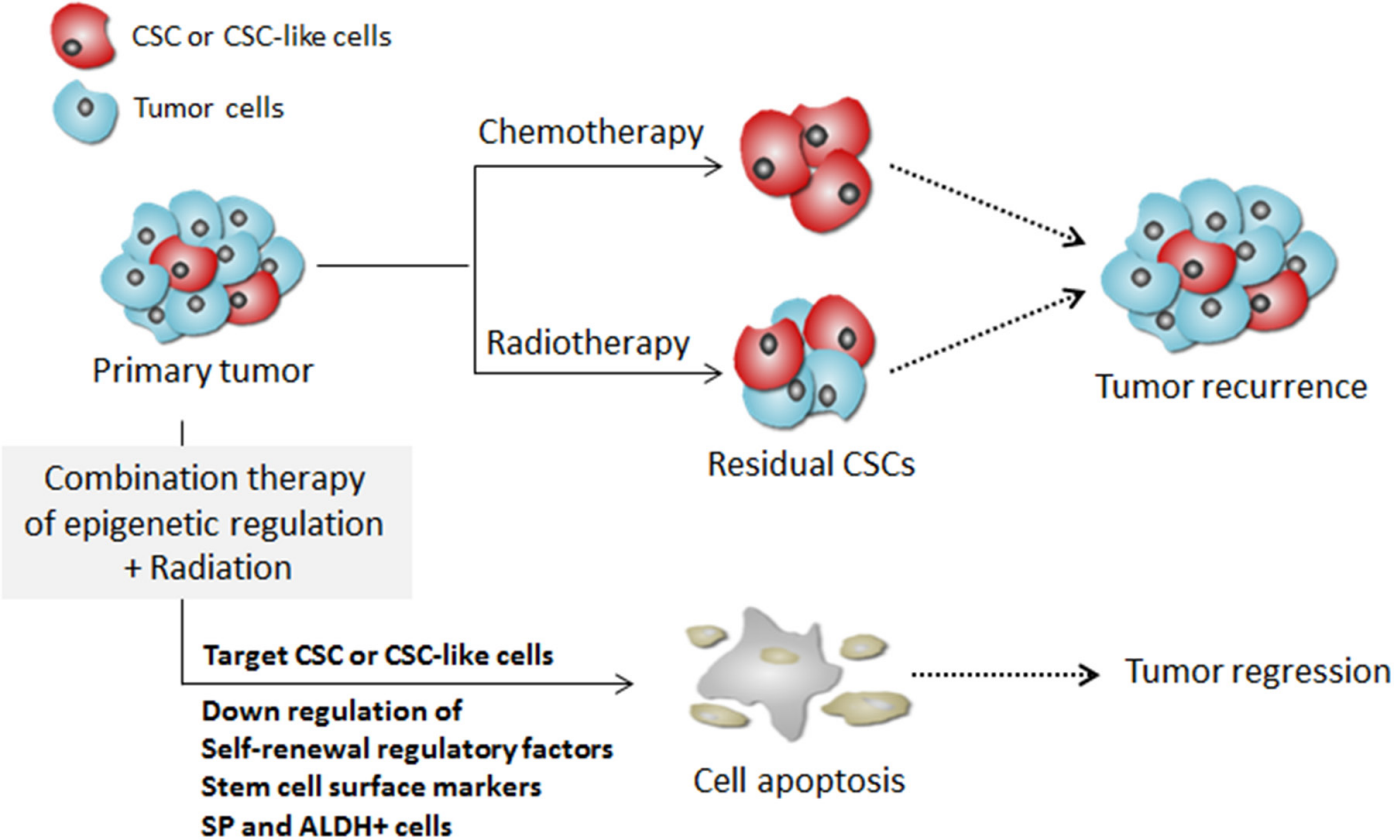
A schematic model illustrating the targeting of CSCs by combination treatment of DNA methylation inhibitor with radiation

A schematic model illustrating the possible mechanism of targeting pancreatic CSCs by the combination treatment with 5-aza-dC and IR is given in Figure 7. Tumors consist of both highly proliferative cancer cells and putative CSCs. Conventional chemotherapy or radiotherapy exert their cytotoxic effect primarily on the highly proliferative tumor cells. However, CSCs expressing high levels of regulatory factors of self-renewal or cell surface markers are usually resistant to conventional cancer therapies including radiotherapy and chemotherapy. Our data showed that combination treatment with an epigenetic inhibitor and radiation targets and eliminates pancreatic CSCs by decreasing the expression of regulatory factors self-renewal and surface markers, as well as EMT-associated molecules. Therefore, the combinatorial use of DNA methylation inhibitors with radiation can eradicate the whole tumor mass by killing both the highly proliferative cells and the CSCs.

In conclusion, we demonstrated that 5-aza-dC, a DNA methylation inhibitor, in combination with IR in pancreatic cancer cells showed enhanced efficacy in inducing apoptosis at both cellular and molecular levels. We also found that the combination treatment with 5-aza-dC and IR effectively downregulated key regulatory factors of CSCs such as Oct4, Sox2, and Nanog, CSC surface markers such as CD44, ALDH1, as well as the proportion of SP cells in a sphere culture system. Therefore, this treatment effectively eliminates CSCs or CSC-like populations and decreases their ability of self-renewal *in vitro* and *in vivo*. Genome-wide transcriptome profiling of pancreatic cancer cell lines treated with 5-aza-dC, IR, and their combination identified epigenetically regulated genes as well as global transcriptional changes of Oct4-driven target genes. These findings suggest that 5-aza-dC in combination with radiotherapy may improve cancer therapy by targeting pancreatic CSCs.

## MATERIALS AND METHODS

### Cell culture, drug treatment and irradiation

Human pancreatic cancer cell lines (MIA PaCa-2 and PANC-1) were obtained from American Type Culture Collection (ATCC, VA, USA). Cells were cultured at 37 °C in 20% O_2_ and 5% CO_2_ in Dulbecco’s Modified Eagle’s medium (Welgene, Korea) containing 10% fetal bovine serum (HyClone, UT, USA) and 1% antibiotic-antimycotic (Gibco, NY, USA). Cells were treated with 5-aza-dC (5 μM) (Sigma, MO, USA) once daily for 3 days. The effect of metformin on the radiation-induced death of cancer cells was determined. Cells were incubated with 5-aza-dC for 3 days before and 24 hours after irradiation, and their clonogenic survival was determined. Cells treated with 5-aza-dC for 3 days without exposure to radiation or those exposed to only irradiation were used as controls. Irradiation (gamma-rays) of cells was performed with a ^137^Cs ray source (Eckert & Ziegler, Germany) at a dose rate of 2.6 Gy/minute.

### Clonogenic survival of cancer cells

Cells (MIA PaCa-2 and PANC-1) were seeded in 6-well plates (5000 cells/well) and treated with 5-aza-dC/irradiation. The cells were cultured for 14 days. The culture medium was replaced every 2 days. The colonies were fixed and stained with 1.25% crystal violet, washed extensively to remove excess dye and imaged using a Samsung scanner. To quantify the number of colonies, colonies with >50 cells were counted.

### Cell proliferation assay and cell viability assay

Cell proliferation was determined using MTT assay for adherent cells and WST-1 assay for sphere cells. Cells (2 x 10^5^ cells/well) were seeded in 6-well plates and incubated at 37°C. After 48 hours, the cells were washed twice with PBS, and 5 mg/ml MTT or WST-1 solution in PBS was added to each well for 4 hours. After removal of the each solution, a solubilization solution (DMSO/EtOH, 1:1 ratio) was added to each well to dissolve the formazan crystals. The absorbance range from 440 to 500 nm was measured using a Paradigm microplate reader (Beckman Coulter, CA, USA). Cell growth curves: MIA PaCa-2 and PANC-1 cells treated with 5-aza-dC and/or irradiation were seeded at a concentration of 50,000 cells/well in 6-well plates. After incubation for 24, 48, 72 and 96 hours, the cells were harvested, diluted with trypan blue working solution and counted for the construction of growth curves.

### Tumor growth analysis

Animal experiments were performed in accordance with the guidelines for the use of laboratory animals and approved by the Institutional Animal Care and Use Committee of DIRAMS (DI-2016-018). Human pancreatic tumor xenografts were established in 6-week-old female SCID mice. The SCID mice were obtained from Japan SLC (Japan). MIA PaCa-2 cells (1 x 10^6^ cells) were subcutaneously inoculated into the flanks of SCID mice. To measure tumor xenografts, the mice were randomly divided into the following 6 groups: (1) mock, (2) 5-aza-dC, (3) IR 2 Gy, (4) IR 4 Gy, (5) 5-aza-dC plus irradiation (2 Gy) (6) 5-aza-dC plus irradiation (4 Gy). The tumor volumes were measured once per week. Tumor volume (V) was monitored by measuring the length (L) and width (W) of the tumor with calipers and was calculated with the formula V = (L x W^2^) × 0.5. The mice were sacrificed after 5 to 7 weeks (Figure 1D and 4E) and the tumors were removed. Approximately 5 x 10^5^ sphere-forming MIA PaCa-2 cells in 200 μl of sphere growth medium were subcutaneously injected into the flanks 6-week-old female SCID mice. The xenografts formed from the sphere-forming cells were assessed using the methods described above.

### Flow cytometric analysis

Cells from both adherent and sphere cultures were dissociated into single cells, washed and suspended in PBS. For the identification of cell surface markers, the cells were labelled with anti-CD24, anti-CD44, anti-CD133, or anti-ALDH1 and secondary fluorescein (FITC)- or APC-conjugated antibodies (BD Pharmingen, CA, USA). Flow cytometry was performed on a FACScan system (BD FACSAria™, BD Biosciences, CA, USA). Annexin V for apoptosis analysis: Apoptosis in treated cells was assessed using an Annexin V/FITC Apoptosis Detection kit (BD Bioscience, MA, USA). The cells were stained with PE, Annexin V and 7-amino-actinomycin (7-AAD) and incubated for 15 min in the dark.

### Western blot analysis

Cells were lysed in lysis buffer. Equal amounts of total proteins were loaded onto 4-12% SDS–PAGE gels and transferred to PVDF membranes (GE Healthcare Life Sciences, NJ, USA). The membranes were blocked with 5% milk dissolved in TBS containing 0.02% Tween 20 and incubated overnight at 4°C with specific primary antibodies. The membranes were subsequently incubated with specific horseradish peroxidase-conjugated secondary antibodies. Protein bands were visualized using a Fusion FX5 system ((Vilber Lourmat, France). The following primary antibodies were used: anti-cleaved caspase 3, anti-E-cadherin, anti-cleaved caspase 9, anti-cleaved PARP1 (Cell Signaling Technology), anti-SNAIL1, anti-Vimentin, anti-Twist, anti-Slug, anti-Zeb1, anti-Nanog, anti-Sox2, anti-CD44 (Santa Cruz), anti-N-cadherin and anti-Oct4 (BD Biosciences), anti-Survivin, anti-CD133 (Abcam), anti-ALDH (Avivasysbio), and anti-β-actin (Sigma).

### Aldefluor assay

Sphere-forming cells from pancreatic cancer cell lines (MIA PaCa-2 and PANC-1) were treated with 5-aza-dC for 3 days and 24 hours after irradiation and washed. The cells were then dispersed into single cells, and the ALDH activity was determined with Dry ALDEFLUOR™ Reagent (STEMCELL Technologies, Canada) by following the manufacturer’s instructions.

### Side population assay

Sphere-forming cells from pancreatic cancer cell lines (MIA PACa-2 and PANC-1) were treated with 5-aza-dC for 3 days and 24 hours after irradiation and washed. The cells were then dispersed into single cells, incubated with Hoechst 33342 (Sigma) at 50 mmol/mL either alone or in combination with the ABC transporter inhibitors verapamil (50 mmol/mL) (Sigma) and FTC (10 mM) for 90 min at 37 °C. After being washed, the SP cells were identified using flow cytometry.

### Sphere-forming assay

MIA PaCa-2 and PANC-1 cells were plated in ultra-low attachment 6-well plates (Corning, NY, USA) at a density of 5,000 cells/well. The cells were maintained in serum-free DMEM/F12 supplemented with 2% B27 supplement (Gibco, MA, USA), 1% N2 supplement (Gibco), 20 ng/ml epidermal growth factor, EGF (Gibco), 20 ng/ml fibroblast growth factor, FGF (Gibco), and 1% antibiotic-antimycotic (Invitrogen, CA, USA). The number and size of tumor spheres formed were evaluated by light microscopy after 7-12 days.

### Caspase 3/7 activity and comet assay

Cells (5 x 10^3^) were seeded with 100 μl DMEM on 96-well plates. After 24 hours of incubation, 100 μl substrate and buffer mixture from a Caspase-Glu 3/7® Assay Kit (Promega, Madison, USA) was added to each well. After gentle mixing for 30 min, the plates were incubated at room temperature for 1 hour in the dark. Fluorescence activity was measured with a luminometer (ATTO, Japan). All procedures were repeated 3 times and included a negative control. Comet assays for DNA damage analysis were performed with an OxiSelect™ Comet Assay Kit (Cell Biolabs, CA, USA) by following the manufacturer’s instructions. Comet tails were detected using a fluorescence microscope (Nikon, Japan).

### Migration and invasion assay

Cell migration was determined using Transwell plates (24-well, 8 μm pore size, Corning Costar, NY, USA), and invasion assay was carried out by using Matrigel-coated invasion chambers (24-well, 8 μm pore size, Corning Costar). The upper chamber contained pancreatic cancer cells in serum-free medium and the lower chamber contained DMEM medium supplemented with 10% FBS. Cells were incubated for 16 hours at 37°C in atmospheric conditions of 20% O_2_ and 5% CO_2_. The non-migrated cells were scraped off from the upper surface of the membrane with cotton swabs. The migrated cells remaining on the bottom surface of the membrane were counted after staining with Giemsa (Sigma). Photographs were taken using a QIcam image camera system mounted on a Nikon ECLIPSE 80i microscope (Nikon).

### RNA sequencing

RNA-seq was performed for MIA PaCa-2 and PANC-1 cells treated with 5-aza-dC or IR exposure (2 Gy and 4 Gy) and both combination. Briefly, total RNA was extracted from MIA PaCa-2 and PANC-1 cell lines with different conditions. For the Illumina library preparations, the extracted RNAs was synthesized using TruSeq RNA sample preparation kit (Illumina Inc., CA, USA) and sequenced using Illumina HiSeq 2000. Paired-end reads were trimmed by quality score using Trimmomatic [51] and mapped to the human reference genome (hg38) using HISAT2 [52]. Gene expression levels were quantified by means of the reads per kilo base per million mapped reads (RPKM) method. Differentially expressed genes between different conditions were identified using the GFOLD algorithm [53]. Lowly expressed genes which were below 1 RPKM were discarded.

### Statistical analysis

The results are presented as the mean ± standard deviation. Statistical analyses were performed using Student’s *t*-test. *P*-values of < 0.05 were considered significant.

## SUPPLEMENTARY MATERIALS FIGURES AND TABLES




