# Supplementary material for: Combinatorial effects of an epigenetic inhibitor and ionizing radiation contribute to targeted elimination of pancreatic cancer stem cell
Source: Oncotarget. 2017 Oct 6;8(51):89005–20. doi: 10.18632/oncotarget.21642 (PMC5687664; doi:10.18632/oncotarget.21642)
Supplement: Supplementary file 1 [file oncotarget-08-89005-s001.pdf]

Combinatorial effects of an epigenetic inhibitor and ionizing radiation contribute to targeted elimination of pancreatic cancer stem cell

SUPPLEMENTARY MATERIALS

Bisulfite modification and quantitative methylation-specific PCR (qMSP)

Genomic DNA from pancreatic cancer cells was isolated using by a Phenol/Chloroform method. Bisulfite modification of 2 µg genomic DNA was performed using EZ DNA Methylation kit (Zymo Research). For positive control and negative control, *in vitro* methylated DNA (IVD) and H_2O were treated, respectively. For methylation analysis of target genes, quantitative MSP amplification was performed on bisulfite treated samples and normalized by *Alu* element. All primer designed using MethPrimer (http://www.urogene.org/cgi-bin/methprimer/methprimer.cgi) and presented in Supplementary Table 1. Real-time RT-PCR was performed by a CFX96™ real-time system (BioRad).

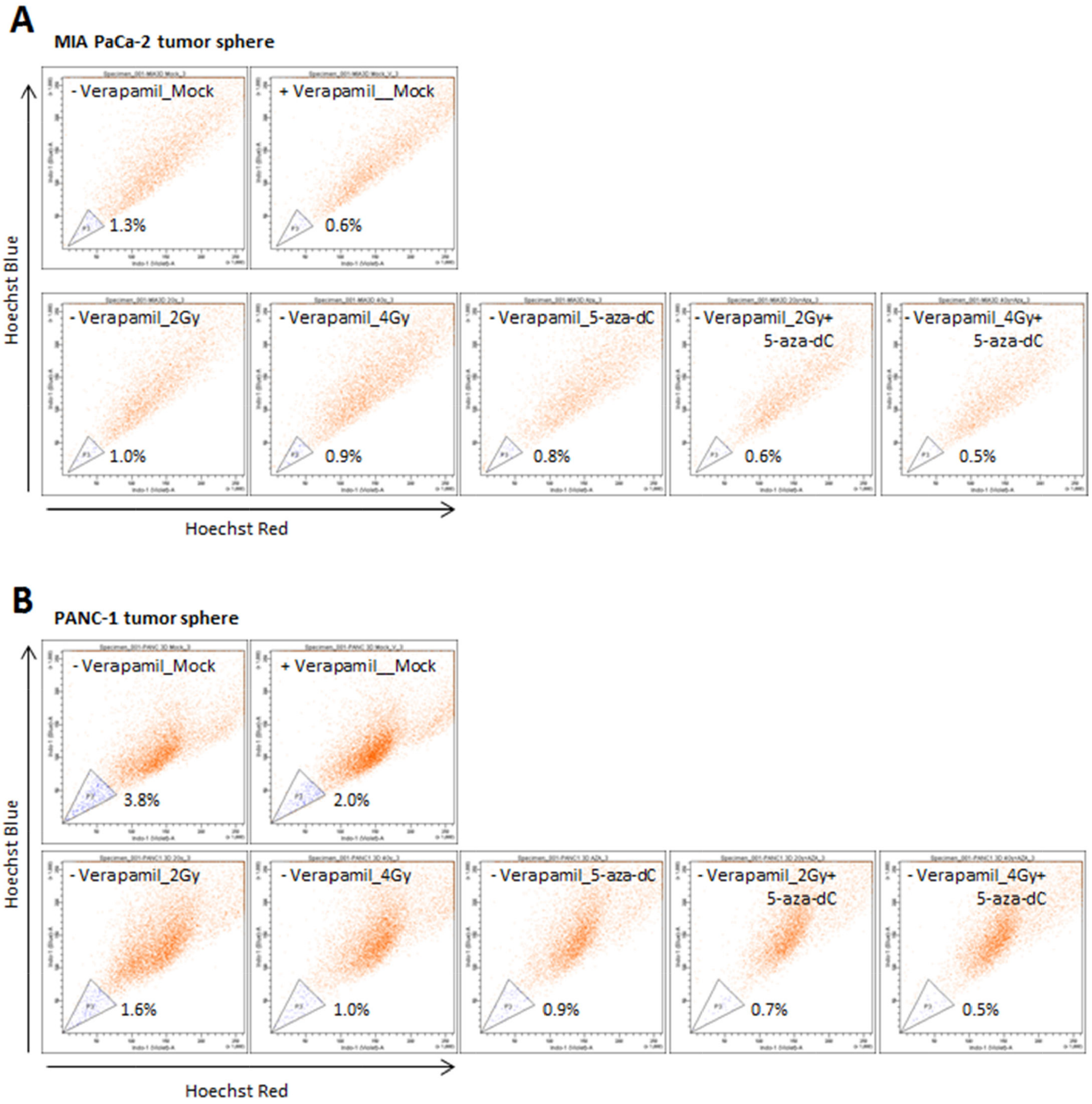

Supplementary Figure 1: Effects of 5-aza-dC in combination with IR on pancreatic CSCs. Changes in percent of side population (SP) in sphere cultured cells from MIA PaCa-2 **(A)** and PANC-1 **(B)** treated with 5-aza-dC alone or with IR. Cells were stained with Hoechst33342 dye in the absence or presence of verapamil (50 uM) and analyzed by flow cytometry. The representative images were depicted from 3 independent experiments.

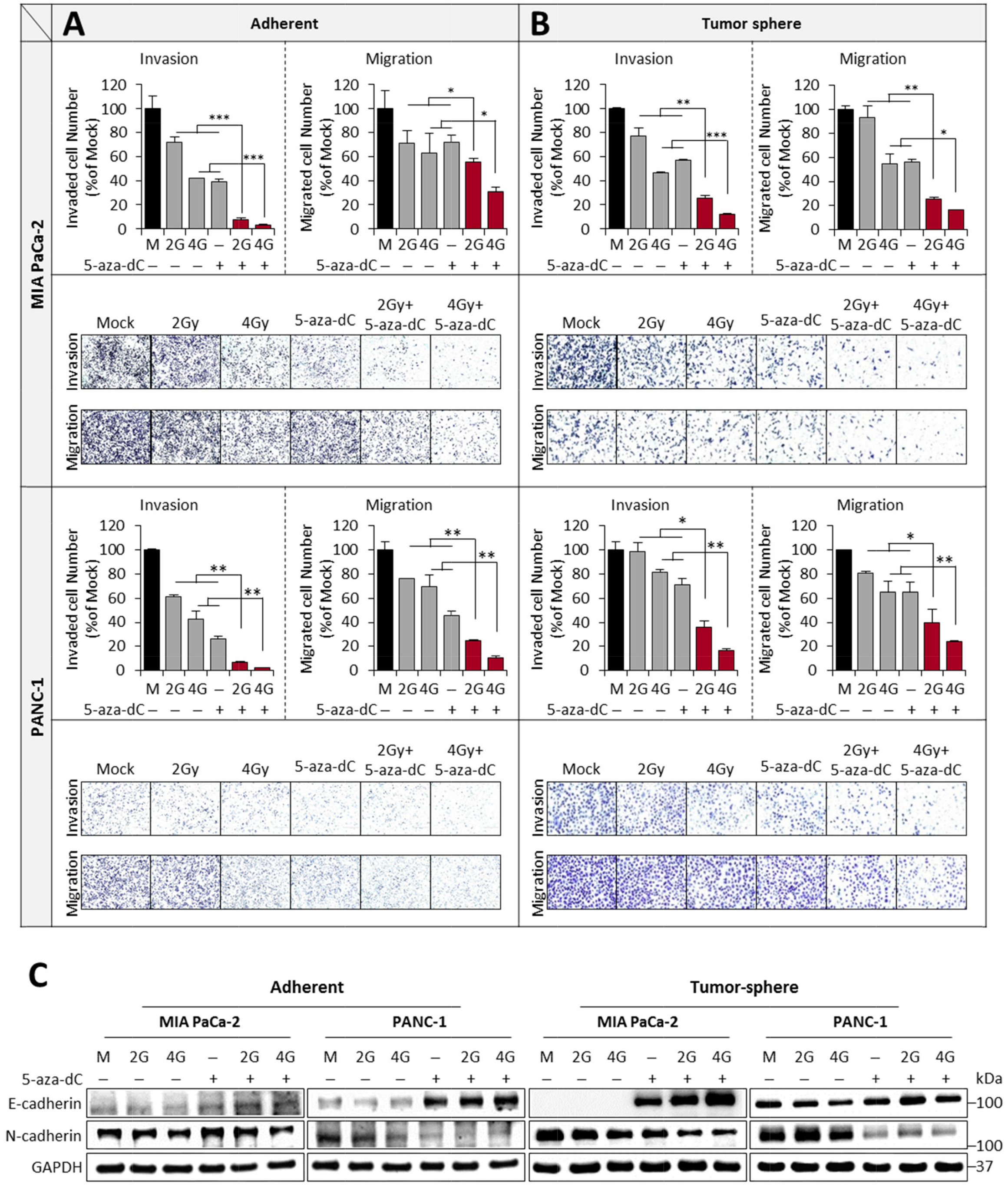

Supplementary Figure 2: Combination treatment of 5-aza-dC with IR contributes to suppress invasive traits. Migration and invasion assays in MIA PaCa-2 and PANC-1 adherent cells **(A)** and sphere forming cells **(B)**. **(C)** Immunoblot analysis of E-Cadherin and N-Cadherin expression in both adherent pancreatic cancer cells and sphere forming cells. GAPDH protein level s served as loading controls. Data are means ± SD from 3 independent experiments. *P*-values were calculated using Student's *t*-test. *P<0.05; **P<0.01.

Supplementary Table 1: PCR primer information for experiment

Representative genes	Experiment		Primer sequences (5'-3')	
			Forward	Reverse
**MT1X**	MSP	Unmethylation	GATTGTTTTGTAAGGGTAATTATGT	CCAAACAACTTATTAAACTCCTCATC
		Methylation	GATTGTTTCGTAAGGGTAATTACGT	CCAAACAACTTATTAAACTCCTCGT
**TRNP1**	MSP	Unmethylation	TATTGGTTGGGAGGAGTAAATATGA	AAAAAAATTAACACAAAAAAAACAAC
		Methylation	AATATCGGTTGGGAGGAGTAAATAC	AAAAAATTAACGCAAAAAAACGAC
**MT1G**	MSP	Unmethylation	GTAGGGTGAGAGAAGTTGTATATGG	TTTTATAATCAAAAAACAAAACCAAA
		Methylation	TGGTAGGGTGAGAGAAGTCGTATAC	TTTTTATAATCAAAAAACGAAACCG
**RAB38**	MSP	Unmethylation	AGGTTGTGTTTTTTTGGTTAGGTAT	TAATAATACTAATCTTCCCCACACC
		Methylation	GTTGCGTTTTTTTGGTTAGGTAC	TTAATAATACTAATCTTCCCCACGC
**Alu**		Normalization	TGGTTAATATGGTGAAATTTTGTTTTTATT	TCCTACCTCAACCTCCCAAATAACT

Supplementary Table 2: Gene list of heatmaps in Figure 6

See Supplementary File 1
