# Supplementary material for: Combinatorial effects of an epigenetic inhibitor and ionizing radiation contribute to targeted elimination of pancreatic cancer stem cell
Source: Oncotarget. 2017 Oct 6;8(51):89005–20. doi: 10.18632/oncotarget.21642 (PMC5687664; doi:10.18632/oncotarget.21642)
Supplement: Supplementary file 2 [file oncotarget-08-89005-s002.docx]

**Supplementary Table 2:** **Gene list of heatmaps in Figure 6**

| **MIA PaCa-2 (Fig. 6A)** | **PANC-1 (Fig. 6B)** | **Oct4 target genes (Fig. 6c)** |
| --- | --- | --- |
| RPSAP9 | HIST2H3D | TRAP1 |
| COL1A1 | SPHAR | MYBL2 |
| TRNP1 | EFNA2 | PSMA6 |
| HIST1H2AK | RAD51-AS1 | HNRNPUL1 |
| LOC105373378 | RGCC | CCND1 |
| RPL13AP20 | SLC29A4 | PRKDC |
| LINC01420 | GPR137C | MKI67 |
| SCARNA27 | KCNS3 | PLEC |
| KRT17 | RAET1E-AS1 | SDC1 |
| LOC100507437 | GIPC3 | ZNF217 |
| SCARNA16 | ZDHHC2 | CAD |
| CTRL | CT45A3 | GSK3A |
| GPX7 | GPR75 | MYO18A |
| LOC101929089 | ELDR | LAMB1 |
| GLRX | SCARNA11 | AP2A2 |
| RAB38 | LRRC75B | C19orf12 |
| LOC105376854 | TMEM81 | LANCL2 |
| VPS13A-AS1 | LOC105371814 | PTEN |
| MT1X | FOXE3 | SLC39A13 |
| LINC00239 | SOCS1 | NIPBL |
| LOC100288866 | ZNF219 | SLC12A4 |
| LOC102724532 | RFLNA | SLC29A4 |
| HIST1H3B | TIGD5 | SMYD3 |
| RNU7-1 | VSTM2L | C2CD5 |
| MT1G | PRRX2 | MTR |
| LGALS7B | TMEM52 | TRIP11 |
| LOC100288069 | IRX5 | AFAP1 |
| LIPT2 | SLC38A5 | CHMP2B |
| LINC00115 | LYPD5 | AGO2 |
| GAGE12H | MMP25 | MAT1A |
| TCEB3CL | LOC101928123 | MUC5B |
| PARD6G-AS1 | USP46-AS1 | SLC7A9 |
| MT1E | LOC105375304 | MUC6 |
| LOC101927365 | LOC100129046 | MYOM2 |
| SUSD3 | A4GALT | FGF4 |
| RPS21 | TIGD3 | SLC9A9 |
| MT1M | IGSF9 | KCNK9 |
| TFPI2 | LOC102723704 | NTM |
| MT2A | GPRIN2 | ZBTB16 |
| TCEB3C | NPAS1 | FGF3 |
| NUDT17 | SLCO4A1-AS1 | PRR21 |
| GAGE12C | HCN2 | IL21R |
| GAGE12E | ZSWIM4 | MUC17 |
| BBOX1-AS1 | TMEM173 | CSMD1 |
| NINJ2 | ZNF703 | CASC1 |
| SDSL | SEMA6B | MUC2 |
| BIK | ZNF358 | PDZD2 |
| LOC100506639 | NXPH2 | PTPRE |
| NKILA | FAM117A | LILRB2 |
| SPINT1 | CILP2 | ADAMTS17 |
| PHLDA2 | MBLAC1 | LILRB5 |
| B4GALT1-AS1 | ADM2 | SOX11 |
| SLC7A5P2 | DCHS1 | FAM153A |
| CXCL2 | PGAM4 | ADAMTS2 |
| SCARNA9 | FAM131C | COL23A1 |
| GPRC5D | RPL13P5 | DBH |
| LOC101927787 | RNF208 | RYR2 |
| LINC01336 | PTPRU | PAX6 |
| SCARNA11 | PAK6 | COL17A1 |
| SCARNA13 | C8orf46 | STAG3 |
| FFAR1 | NRROS | TERT |
| NKX2-6 | MEIOC | RASA4 |
| C17orf100 | TCEB3C | CDH15 |
| ANKRD37 | SP6 | ZDHHC11 |
| EME2 | WNK2 | FOXO4 |
| RAB42 | TCEB3CL | GLDC |
| LOC105369980 | B3GNT6 | FBXO31 |
| NAV2-AS2 | FCGBP | FRG1 |
| MINCR | KCNF1 | EOMES |
| NUDT18 | GDPGP1 | SIRPA |
| ADSSL1 | TBX2 | DTNBP1 |
| IL1B | OLFML2B | HDAC4 |
| HAP1 | TMEM38A | TRAPPC9 |
| SCARNA8 | LOC100996583 | JARID2 |
| HIST1H2AM | SYNGR3 | PRX |
| RUSC1-AS1 | DISP2 | VASH1 |
| CARD9 | P4HA2-AS1 | PCSK6 |
| ALDH2 | LOC100289580 | DKK3 |
| LOC389641 | KCNQ4 | PRSS3 |
| RIBC2 | GPR135 | OPRD1 |
| ZNF789 | SOX18 | GPIHBP1 |
|  | NAV2-AS2 | TNC |
|  | FZD9 | RP1L1 |
|  | GDPD5 |  |
|  | PRR7 |  |
|  | SCARF2 |  |
|  | LOC102724776 |  |
|  | LOC100289361 |  |
|  | BBC3 |  |
|  | GCSHP3 |  |
|  | HIST1H3E |  |
|  | TRMT13 |  |
|  | UCN2 |  |
|  | METTL25 |  |
|  | PID1 |  |
|  | C1orf53 |  |
|  | LOC105372672 |  |
|  | MT3 |  |
|  | KCNG3 |  |
|  | CEP85L |  |
|  | KDM7A |  |
|  | PGBD3 |  |
|  | SUGT1P1 |  |
